# Neurology and Sulphuric Ether

**Published:** 1848-01

**Authors:** J. F. B. Flagg


*Neurology and Sulphuric Ether.* By J. F. B. Flagg, M.D.

The theory of the *nervous system*, or rather that portion which embraces the passage of the nerves from the brain to the outer surfaces of the body, has heretofore been held to consist merely in a double arrangement of organization, thus classified: 1st, The *motor nerves*, or those which incite to action; and 2d, The *sensor*, or that complicated system of machinery which is so constructed as to convey to the mind various sensations through one medium. One set of nerves has been thought sufficient to allow us to discriminate between that sensation which we denominate simply *touch* or *feeling*, and that commonly called *pain.*

From a series of experiments with sulphuric ether, in which I have been engaged for some time past, I am in possession of *facts*, which certainly go far to refute preconceived opinions touching this interesting question in physiology; yet, with proper diffidence would I lay before the scientific world, deductions I cannot resist, arising from those facts.

That ether can induce that condition of perfect unconsciousness of pain during the passage of the surgeon's knife in any known operation, is a question at the present moment entirely beyond cavil. As to the propriety or safety of so doing, it is a matter of no importance in reference to this subject of communication, except that it has led to as little use of the article as would justify us in securing the greatest good. For example: Cases have presented themselves to me where I would not suffer more ether to be taken than would cause the *consent* of the patient to the removal of a tooth; a consent that could not be obtained under usual circumstances. The pain probably was as great, or nearly so, as it would have been without the ether; but even under these circumstances, such individuals have been very united in ceding to it great merit.

It therefore became a desideratum (in the dental department particularly) to give, as nearly as possible, just a suitable quantity, and no more; and in my endeavours to establish this point, many peculiar mental and physical effects have been produced, which I have deemed worthy from time to time of presenting to the public through different periodicals of the day.

There is a particular point of etherization, which, if improved at the moment, will leave the patient in full possession of all his faculties, with the single exception of the SENSE OF PAIN; and, particularly, the consciousness of *touch* is as acute as under ordinary circumstances, if not quickened even. To account for this, *I assign to the system two sets of nerves; one, to carry to the brain the idea of* TOUCH—*the other of* PAIN.

I submit the following cases and certificate in support of my theory:—

A lady, operated upon for scirrhous breast, I so etherized as to keep her in conversation upon indifferent subjects through the whole operation, which required two incisions, together about sixteen inches in extent, the entire removal of the gland, and the taking up of five or six arteries. Her replies to my questions were in a drowsy tone of voice, but strictly correct and consistent; her sense of taste and smell were tested by one of the medical gentlemen present, and found to be unaffected. She expressed much astonishment when told that the operation was completed, and observed *that she only felt them marking it out.*

So large a number have told me that they felt distinctly every part of an operation, *unconnected with pain*, that I deem it only necessary to report the following, as illustrative of *increased* sense of touch:—

In extracting a tooth for a gentleman, I used the German key, (an instrument that carries with it many painful associations.) I requested him to note any little thing that might occur to him, and explain it to me after the operation. He said that he was entirely conscious to the sense of touch; so much so, that he could detect, by the feeling, that the *bandage* which I used upon the fulcrum was composed of *silk;* nothing like pain was felt.

Several have remarked that a sensation of *cold* has been imparted from the instrument, without pain.

The following certificate I am authorized to submit:

[Copy.] *Philadelphia, Dec.* 15, 1847.

My attention having been taken by Dr. J. F. B. Flagg to the subject of fixing my mind upon the sensation of *touch* while under the influence of ether, for the purpose of having a tooth extracted, I hereby certify, that at the moment my tooth was removed, I distinctly felt the slightest touch upon my *hand*, my *knee* and my *forehead*. That I felt the whole process of extracting my tooth, but not the least pain. F. Stout.

In reference to the above case, I would mention that *after* the gentleman had completed inhaling, and the instant *previous* to extracting his tooth, I made a cross upon his knee with my finger, so delicately as scarcely to be sensible of the touch myself. Upon the effect of the ether passing off, I asked him what figure I described upon his knee? He replied that he barely felt it, but what he *did* feel was like a figure 8. Now it will readily be perceived, that in order to make a cross, we necessarily make the motion as described by this patient.

